# Early markers of gestational diabetes mellitus: what we know and which way forward?

**DOI:** 10.11613/BM.2021.030502

**Published:** 2021-10-15

**Authors:** Jelena Omazić, Barbara Viljetić, Vedrana Ivić, Mirta Kadivnik, Lada Zibar, Andrijana Müller, Jasenka Wagner

**Affiliations:** 1 Department of Laboratory and Transfusion Medicine, National Memorial Hospital Vukovar, Vukovar, Croatia; 2 Department of Medical Chemistry, Biochemistry and Clinical Chemistry, Faculty of Medicine, J.J. Strossmayer University, Osijek, Croatia; 3 Department of Medical Biology and Genetics, Faculty of Medicine, J.J. Strossmayer University, Osijek, Croatia; 4 Clinic of Obstetrics and Gynecology, University Hospital Center Osijek, Osijek, Croatia; 5 Department of Obstetrics and Gynecology, Faculty of Medicine, J.J. Strossmayer University, Osijek, Croatia; 6 Department of Pathophysiology, Faculty of Medicine, J.J. Strossmayer University, Osijek, Croatia; 7 Department of Nephrology, Clinical Hospital Merkur, Zagreb, Croatia

**Keywords:** gestational diabetes, pregnancy, biomarkers, diagnosis

## Abstract

Women’s metabolism during pregnancy undergoes numerous changes that can lead to gestational diabetes mellitus (GDM). The cause and pathogenesis of GDM, a heterogeneous disease, are not completely clear, but GDM is increasing in prevalence and is associated with the modern lifestyle. Most diagnoses of GDM are made *via* the guidelines from the International Association of Diabetes and Pregnancy Study Groups (IADSPG), which involve an oral glucose tolerance test (OGTT) between 24 and 28 weeks of pregnancy. Diagnosis in this stage of pregnancy can lead to short- and long-term implications for the mother and child. Therefore, there is an urgent need for earlier GDM markers in order to enable prevention and earlier treatment. Routine GDM biomarkers (plasma glucose, insulin, C-peptide, homeostatic model assessment of insulin resistance, and sex hormone-binding globulin) can differentiate between healthy pregnant women and those with GDM but are not suitable for early GDM diagnosis. In this article, we present an overview of the potential early biomarkers for GDM that have been investigated recently. We also present our view of future developments in the laboratory diagnosis of GDM.

## Introduction

During pregnancy, women’s metabolism undergoes numerous changes with respect to carbohydrates, fats and proteins in order to provide the nutrients and oxygen needed for foetal growth, and to fill the extra energy stores required for delivery and lactation (*1*). Major changes in any of these metabolic pathways can lead to gestational diabetes mellitus (GDM). Gestational diabetes mellitus is an independent type of diabetes defined as glucose intolerance, with first recognition arising during pregnancy (mostly in the second trimester between the 24th and 28th week of gestation) and resolving after pregnancy.

Today 1-36% of pregnant woman suffer from GDM, depending on the population and criteria used. The incidence of GDM increases along with increases in the prevalence of obesity and diabetes mellitus type 2 (T2DM), which are most commonly associated with the modern lifestyle (*2*, *3*).

The cause and pathogenesis of GDM, a heterogeneous disease, are not completely clear; they could relate to genetic alteration, the deregulation of placental hormones, or ß-cell injury (similar to type 1 diabetes) (*4*). Pregnancy is a state involving metabolic changes, in which the mothers’ physiological systems adjust to enable normal foetal development. In a healthy pregnancy, insulin insensitivity caused by maternal and placental hormones (prolactin, estrogen and cortisol) gradually develops, with a reciprocal increase in insulin secretion of 200% to maintain euglycaemia, and insulin sensitivity decreases by as much as 70% (*5*-*7*). Gestational diabetes mellitus is a pathophysiological state in which there is insufficient insulin available to maintain glucose concentrations in normal range, and hyperglycaemia occurs. Glucose control in pregnancy depends on ß-cell insulin secretion, the insulin clearance required to maintain the balance of hormonal changes in pregnancy, and insulin actions in the liver, muscles and tissues. Disruption in any of these factors leads to insulin resistance during pregnancy and increased serum glucose (*8*, *9*).

**Figure 1:**
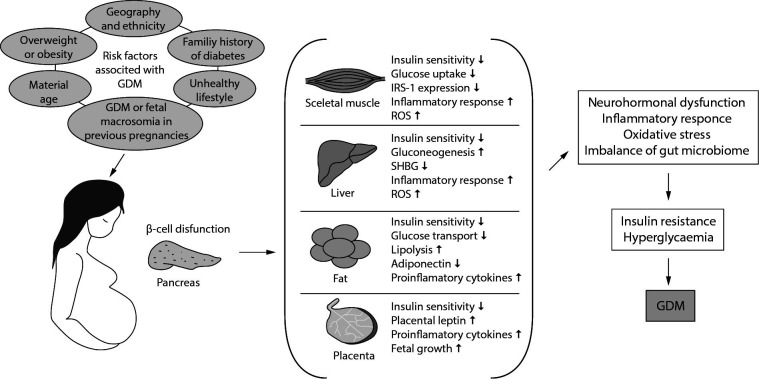
Pathophysiological events specific to gestational diabetes mellitus (GDM). IRS-1 - Insulin receptor substrate 1. ROS - reactive oxygen species. SHBG - sex hormone-binding protein.

In addition, pregnancy is a proinflammatory condition, and the immune response of the maternal immune system during pregnancy is not well regulated under GDM conditions. A common symptom of hyperglycaemic conditions, including GDM, is oxidative stress, which induces an inflammatory response and increases insulin resistance. Oxidative stress also affects the proliferation, activation, cytokine secretion and damage of cellular components (*10*, *11*). Inflammatory cytokines and insulin post-receptors interact to block the normal tyrosine phosphorylation of the insulin receptor substrate, reducing its ability to bind the insulin receptor and thus affecting the regulation of glucose metabolism (*12*). Pathophysiological events specific to GDM are presented in Figure 1.

Gestational diabetes mellitus complications involve intrauterine metabolic alterations in infants, and impact foetal programing, with long-term consequences later in life (*13*). Gestational diabetes mellitus has short- and long-term implications for the mother as well. For the mother, the short-term consequences include hypertensive disorders in pregnancy, failures in progress in labour, the requirement of caesarean section, pre-term delivery, and preeclampsia. The long-term implications are associated with recurrent GDM in subsequent pregnancies, T2DM, and cardiovascular diseases later in life. The short-term implications for the child include macrosomia, perinatal death, neonatal hypoglycaemia, shoulder dystocia and related birth injures, hyperbilirubinemia, and a low Apgar score (*2*, *14*). The long-term implications for the child are T2DM, obesity, and GDM (females only) (*7*). As such, it is important to find early biomarkers that will help to predict GDM and initiate the appropriate treatment to prevent complications. The main challenge is to find a good biomarker for early diagnosis of GDM, which requires having a truly representative sample, and the determination of the exact cut-off point to distinguish healthy pregnant women from those with GDM. Any method that is inexpensive, readily available, automated, and provides results quickly would be welcomed, as would the derivation of biomarkers via minimally invasive methods.

## Methods

With the aim to provide an overview of the early biomarkers of GDM that have recently been published, we have searched PubMed, Ovid MEDLINE and Web of science databases between January and March 2021. Keywords used were: gestational diabetes, biomarkers, GDM risk, inflammation, GDM amino acid biomarker, amino acid profiles in GDM and metabolism or immunology. Articles, regardless of the time frame, in which analysed sample was blood or urine were included, while articles that discuss treatment of GDM were excluded from this review. The articles included are original articles with the results of clinical studies or review articles. In addition to articles found in the databases, the guidelines from national societies used in routine GDM diagnostics were used to write this review.

## Diagnosis of GDM

Most diagnoses of GDM are made according to the guidelines from the International Association of Diabetes and Pregnancy Study Groups (IADSPG), which involve performing an oral glucose tolerance test (OGTT) between 24 and 28 weeks of pregnancy. In 2010, the Hyperglycemia and Adverse Pregnancy Outcome (HAPO) study established new thresholds for OGTT determination between 24 and 28 weeks of pregnancy: 5.1 mmol/L for fasting plasma glucose, 10 mmol/L for one-hour plasma glucose and 8.5 mmol/L for two-hour plasma glucose. The World Health Organization (WHO) has accepted these thresholds, and these guidelines have also been accepted by the Croatian Society for Gynecology and Obstetrics and the Croatian Chamber of Medical Biochemists (*15*-*17*). The National Institute of Health (NIH) suggests a two-step diagnostic test between the 24th and 28th weeks of gestation: a 1-hour screening test with 50 g of glucose for all pregnant women, and then a 100 g OGTT for women who meet or exceed the screening threshold for a blood glucose concentration of 7.2, 7.5 or 7.8 mmol/L in the first step (*18*, *19*).

An alternative test uses glycated haemoglobin (HbA1c), which has less interlaboratory variation, less intraindividual variability, and is not affected by meals, fasting, acute stress or medications (*6*). However, no thresholds have been established for HbA1c in pregnancy; in four studies, different thresholds (5.0, 5.3, 5.5 and 7.5%, respectively) were estimated for GDM (*20*-*23*). However, most guidelines recommend HbA1c > 6.5% for the diagnosis of GDM (*24*).

Existing international guidelines recommend testing for pregestational diabetes in women in early pregnancy with body mass index (BMI) > 30 kg/m^2^, impaired glucose tolerance, and a history of GDM (*7*).

## Conventional predictive GDM biomarkers

Fahami *et al.* and Riskin-Mashiah *et al.* showed that fasting glucose concentrations above 5 mmol/L in early pregnancy were useful for the prediction of GDM (*25*, *26*), while Donovan *et al.* reported fasting glucose concentrations over 4.7 mmol/L as a threshold for GDM (*27*). Li *et al.* recently reported results of their study which included 2112 pregnant women and concluded that a fasting glucose concentration over 4.5 mmol/L during the first trimester offers optimal specificity and sensitivity for GDM prediction (*28*). The fasting plasma glucose concentration was more accurate than other traditional risk factors, such BMI or age, when used for GDM prediction, but it was less accurate than OGTT testing (*25*, *26*, *29*).

There is evidence to suggest that insulin and C-peptide values are good predictors of GDM. Both insulin measurements-fasting insulin and OGTT insulin (or two-hours-postprandial insulin) showed good sensitivity and specificity (*30*, *31*). The concentrations of fasting insulin and C-peptide are significantly higher in pregnant women with GDM than in healthy pregnant women.

Homeostatic model assessment for insulin resistance (HOMA-IR) is calculated from the values of fasting glucose and fasting insulin or C-peptide. The homeostatic model assessment for insulin resistance has mostly been reported to be significantly more abundant in pregnant women with GDM, and is a good predictor of GDM (*7*). However, some studies have downplayed the significance of HOMA-IR compared to other parameter, or suggested there is no difference between pregnant women with GDM and healthy pregnant women (*14*, *32*). Therefore, it can be concluded that the significance of HOMA-IR is questionable, and HOMA-IR alone is insufficient for use as a predictive marker for GDM.

Sex hormone-binding protein (SHBG) is a glycoprotein produced by the liver. Its production and plasma concentrations are controlled by insulin, estrogen, and progestin. Sex hormone-binding protein has an inverse relationship with insulin resistance, and has therefore been proposed as a predictive marker for GDM. The synthesis of SHBG is stimulated by estradiol, meaning high estrogen concentrations during pregnancy increase SHBG concentrations (*33*, *34*). Testosterone reduces the synthesis of SHBG, and low concentrations of SHBG with high concentrations of testosterone are linked with T2DM. Lower SHBG values before pregnancy and in early pregnancy have been linked with an increased risk of GDM (*5*).

Methods for determining SHBG, C peptide or insulin are immunochemical, which are subject to numerous interferences and reference intervals differ depending on analyser and the reagent used. In order to be able to determine the right cut off value, the large enough sample of the pregnant population is needed for each method. This is difficult and almost impossible to establish because pregnant women are sensitive population, so we believe that markers determined by immunochemical methods are not the best choice for early GDM diagnosis.

Leptin is a hormone produced by the adipocytes, ovaries, and placenta. It regulates energy intake and expenditure, and its levels are increased in obesity. As a result of placental production during pregnancy, leptin concentrations are higher in pregnant than in non-pregnant women (*2*). In GDM, a more significant increase in leptin concentrations is seen, although these data are ambiguous due to the production of leptin in the ovaries and placenta as well.

Adiponectin is a protein produced by adipocytes and has anti-inflammatory and antiatherogenic properties, regulating glucose and fatty acid metabolism (*6*). Unlike leptin, it is not produced by the ovaries and placenta, and does not cross to foetal circulation (*35*). Reduced adiponectin concentrations are associated with obesity, hypertension and T2DM. In pregnancy, the concentrations of adiponectin are reduced most likely as a response to reduced insulin sensitivity (*2*). Previous studies have reported that adiponectin concentrations in the first and second trimesters of pregnancy are lower among women who develop GDM in their third trimester (*5*).

Leptin and adiponectin are most often determined by enzyme-linked immunosorbent assays (ELISA), which means that there are limitations and interferences as in other immunochemical methods. Enzyme-linked immunosorbent assays are often not fully automated and therefore material loss is possible. The advantage of using ELISA in measuring those biomarkers is that the determination of these parameters does not require pregnant women to be fasting.

## Amino acid and fatty acids profiling

Metabolomics studies have shown that branched amino acids (BCAAs) and aromatic amino acids are associated with T2DM and GDM. BCAAs are involved in several metabolic pathways of insulin resistance, and studies have shown that BCAAs reduce insulin secretion through their effects on pancreatic β-cells (*36*). The BCAAs valine, leucine and isoleucine are the most hydrophobic amino acids, and have been linked to obesity, insulin resistance and T2DM. Additionally, several clinical studies using liquid chromatography-mass spectrometry (LC-MS) and targeted nuclear magnetic resonance (NMR) approach have shown these amino acids present at higher concentrations in pregnant women with GDM compared to healthy pregnant women, and they increase in the first trimester, so they can be counted as predictive biomarkers of GDM (*37*, *38*). Using a combination of an NMR metabolome and conventional laboratory assays, White *et al.* reported that concentrations of the BCAAs valine, leucine and isoleucine were higher in the second and the third trimesters of pregnancy in women with GDM compared to healthy pregnant women; unlike the aromatic amino acids phenylalanine and tyrosine, which were elevated only in the third trimester in women with GDM compared to the control group (*39*). Other studies also showed that elevated phenylalanine concentrations increase the risk of GDM (*37*, *38*).

The sulfur-containing amino acids include methionine, cysteine, cystine and homocysteine. They play a role in cellular systems and are associated with vascular disease and cancer. Their concentrations, determined by high-performance liquid chromatography (HPLC) and HPLC-MS/MS, differ in pregnant women with GDM compared to healthy pregnant women; cysteine concentrations are higher in GDM, whereas methionine concentrations are lower, and the results for homocysteine concentrations are conflicting (*18*, *40*, *41*).

In Nevalainen *et al.*’s study, in the first trimester of pregnancy, only the arginine concentrations were higher, while the glycine concentrations were lower in GDM pregnant women compared to the control group analysed by micro mass spectrometry (*42*). Rahimi *et al.* reported that in women with GDM, arginine, glycine and methionine measured by HPLC were present at higher concentrations, and rises in asparagine, threonine, aspartate, phenylalanine, glutamine and arginine increased the risk of GDM (*43*).

Several studies have reported changes in serotonin metabolism in GDM, considering that serotonin is a metabolite of tryptophan, and both play a role in regulating foetal growth and development. There are differences in serotonin concentrations analysed by stable isotope dilution direct-infusion method (SID-MS) and ultra-performance liquid chromatography/hybrid quadrupole time-of-flight mass spectrometry (UPLC-TOF-MS) assays between GDM pregnant women and healthy pregnant women; more precisely, serotonin concentrations are higher in GDM (*44*, *45*).

The literature overview demonstrates that the results from previously published amino acid experiments are contradictory, most likely due to differences in the trimesters of pregnancy at which the research was conducted, the BMI and other comorbidities of the study groups, and the definition of GDM.

Fatty acid and lipid metabolism are intrinsic to the supply of energy needed for the foetus to grow and develop, and to the structural components of cells. Previous studies have suggested there are not many benefits in measuring the routine biochemical parameters of lipid status; increased triglyceride concentrations and lower high-density lipoprotein cholesterol in GDM have been demonstrated, but given their low specificity, these cannot be considered as markers of GDM (*7*, *35*).

In normal pregnancy, the concentrations of foetal fatty acids depend on the maternal concentrations. The proportion of saturated fatty acids (SFAs) increases as the pregnancy progresses, and the foetal concentrations of long-chain polyunsaturated fatty acids (LCPUFAs) depend on maternal intake (*46*). The essential fatty acids omega-3 and omega-6 are precursors of a number of regulatory molecules. Several studies have shown that the placental uptake of LCPUFAs and essential fatty acids were reduced in GDM compared to healthy pregnancies, and that the transfer of SFAs was unaffected (*47*).

Free fatty acids (FFA) could also be responsible for the reduced capacity of pancreatic ß-cells and the elevated insulin resistance in GDM. In 1996, Meyer *et al.* showed that women with GDM had FFA concentration that were more than double those found in healthy pregnant women (*48*).

One study showed that insulin GDM treatment might activate placental insulin receptors protein kinase and mediators of extracellular signal-regulated kinase, leading to an increased expression of fatty acid carriers in the placenta and the adiposity of the foetus. Other studies showed that omega-3 fatty acid supplementation contributes to a better outcome in GDM, a low incidence of hyperbilirubinemia, and a reduced hospital rate in new-borns. Furthermore, the results were even better if vitamin E supplements were taken (*47*).

Analytical methods for measuring amino acids and fatty acids are GC-MS and LC-MS which are demanding and not always available in clinical laboratories. This methods are time consuming and require additional pre-analytical steps which may result in material loss, which are the reasons they did not become routine practice.

## Inflammatory biomarkers

As the inflammatory response is enhanced in GDM, inflammatory markers are probably involved in the development of GDM and could be used as predictive markers. Indeed, numerous studies have shown that inflammatory markers could predict GDM as early as the first trimester of pregnancy (*49*).

The C-reactive protein (CRP) and the highly sensitive C-reactive protein (hs-CRP) are inflammatory markers, and are elevated in pregnancy. Furthermore, pregnant women with GDM show even higher concentrations compared to healthy pregnant women (*2*, *5*, *7*). Since different studies used different methods to assess CRP and different criteria to diagnose GDM, some failed to report elevated CRP in women with GDM (*50*). Furthermore, when BMI is adjusted, the relationship between CRP and GDM becomes weaker or disappears (*51*). Due to the above-mentioned contradictory findings and CRP’s low specificity, it is unlikely to be useful as an independent and specific marker for GDM.

Tumour necrosis factor alpha (TNF-α) and interleukin-6 (IL-6) are the main inflammatory cytokines increased in insulin resistance and GDM. Given their suppression of insulin signal pathways and interference with the anti-inflammatory effects of insulin, they are potential mediators of insulin resistance. Insulin reduces the concentrations of reactive oxygen species produced by mononuclear cells, but this is inhibited by the above-mentioned cytokines (*2*, *9*, *52*, *53*). Both are derived from the placental and maternal immune systems, which are mutually tuned to protect the foetus in the maternal organism. In GDM, the maternal profile remains proinflammatory, and the TNF-α and IL-6 concentrations remain elevated (*49*, *54*). TNF-α and IL-6 are involved in the disruption of insulin signalling and the further stimulation of gene expression related to insulin resistance (*55*). TNF-α acts by inhibiting the tyrosine phosphorylation of the insulin receptor, and thus suppresses insulin signal transduction. IL-6, up to 30% of which is produced by adipose tissue (*56*, *57*), negatively regulates insulin signalling and glucose metabolism in adipocytes, and promotes insulin resistance in liver cells (*47*). A great number of studies have found a positive association between elevated TNF-α and IL-6 in women with GDM. However, other studies have failed to find such significant relationships (*7*, *58*). Since heterogeneity in concentrations of TNF-α and IL-6 was found at different stages of normal pregnancy, it is necessary to further characterize their concentrations at certain time points during pregnancy, which would make TNF-α and IL-6 relevant markers for GDM. What favours the above mentioned markers is that they are determined by automated biochemical (CRP) and immunochemical methods (TNF-α and IL-6) in serum and are applicable to most biochemical and immunochemical analysers due to increasing clinical application in other conditions and could easily be routinely measured for the diagnosis of GDM if future research confirms their clinical value.

## Whole blood parameters

Since inflammatory parameters may indicate an increased risk of developing GDM, numerous studies have tried to find a suitable marker in whole blood.

Erythrocytes have surface-complement receptors, and remove immune complexes from circulation in autoimmune diseases and infections. Yang *et al.* reported that women with GDM had significantly higher values of erythrocytes, and lower mean corpuscular volume and mean corpuscular haemoglobin values (*59*).

Leukocyte number is a biomarker of systemic inflammation. Many studies have reported that women with GDM had significantly higher counts of leukocytes, neutrophils, lymphocytes and monocytes (*11*, *59*). Furthermore, most clinical studies reported that the ratio of these cells is a suitable GDM marker - for example, an elevated neutrophil/lymphocyte ratio (NLR) and monocyte/lymphocyte ratio (MLR) (*11*, *60*-*62*).

Activated platelets release chemokines and P-selectin, and express a variety of membrane receptors involved in inflammation. Yang *et al.* reported that women with GDM displayed significantly higher values of platelets and lower mean platelet volume (MPV) values (*59*). On the contrary, in their clinical studies Kebapcilar *et al.* and Liu *et al.*, women with GDM had higher MPV values associated with platelet aggregation, increased thromboxane A2 and ß-thrombomodulin release, and increased expressions of the receptors of adhesion molecules, which could cause venous occlusion and vasoconstriction (*8*).

However, although blood count is the most available and affordable measurement and the values obtained are comparable among the different manufacturers, these parameters are not of high value since whole blood parameters are nonspecific (both in individuals and ratios) and are found to be altered under various inflammatory conditions. Certain parameters obtained via the newer technologies that are in routine application today, such as flow cytometry, have a higher value.

The immune system is composed of the innate immune system and the adaptive immune system, and T lymphocytes are major components of the immune response. The balance between proinflammatory and anti-inflammatory cells is very important for maintaining the homeostasis of the immune system, and for preventing inflammatory diseases. The T helpers Th1 and Th17 are proinflammatory cells, while regulatory T-cells (Treg) and Th2 are anti-inflammatory cells. In GDM, the ratio of proinflammatory to anti-inflammatory cells changes with increases in Th1 and Th17 cells and reductions in Th2 and Treg cells (*63*). Th1 cells play a pathogenic role by activating macrophages and cytotoxic T-cells, and secrete some interleukins such as IL-2 and interferon gamma (IFN-γ), while Th2 cells induce the production of interleukins (IL-4 and IL-10) that play a protective role during GDM. Th2 cells are also involved in allergic responses by activating the IgE antibody-producing ß-cells (*52*, *64*). Several clinical studies on a small number of respondents have attempted to determine the lymphocyte subpopulations in GDM with flow cytometry, because different cell subpopulations exhibit different clusters of differentiation (CD) molecules. The conclusions of these studies are that in GDM, the concentrations of T-cells (CD3+), T-helper cells (CD3+, CD4+) and cytotoxic T-cells (CD3+, CD8+) are elevated compared to healthy controls (*65*-*67*).

## Novel GDM biomarkers

One routine laboratory test that has shown lower values in GDM compared to a healthy control group is that which assesses brain natriuretic peptide (BNP) (*5*). Some new biomarkers have also arisen with the potential ability to predict GDM. The old and new GDM biomarkers are presented in Table 1.

**Table 1:** Gestational diabetes mellitus predictors and biomarkers

	**Pregnancy period**	**Values in GDM pregnancy**	**References**
**Conventional predictors**			
Impaired glucose tolerance	Between weeks 24 and 28	Assessed by OGTT: fasting > 5.1 mmol/L, 1^st^ hour > 10 mmol/L or 2^nd^ hour > 8.5 mmol/L	15-17
Glycated haemoglobin	NA	> 6.5% (48 mmol/mol)	24
Body mass index	NA	> 30 kg/m^2^	7
History of GDM	NA	positive	7
**Conventional GDM biomarkers**			
Fasting plasma glucose	Early pregnancy	> 5 mmol/L, > 4.7 mmol/L or > 4.5 mmol/L	23,25-27
**Hormones involved in energy homeostasis**			
C-peptide	NA	C-peptide	30,31
Fasting insulin	NA	insulin	30,31
Impaired insulin sensitivity	NA	Deviation from normal insulin concentrations assessed by measurement of fasting insulin and OGTT insulin (or 2 h postprandial insulin)	28
Homeostatic Model Assessment of Insulin Resistance	NA	Insufficient as a predictive marker for GDM	7
SHBG	Before and in early pregnancy	Ż SHBG	5
Leptin	During the whole pregnancy (because of placental production)	leptin	2
Adiponectin	In the first and second trimester	Ż adiponectin among women who develop GDM in the third trimester	2,5
**Amino acid profile**			
Branched amino acids	In the first trimester; also valine, leucine and isoleucine in the second and third trimesters	valine, leucine and isoleucine	36-38
Aromatic amino acids	In the third trimester	phenylalanine and tyrosine	98
Sulfur-containing amino acids	NA	methionine, contradictory findings for cystine and homocysteine	18,40,41
Arginine	In the first trimester	arginine	42
Glycine	In the first trimester	Contradictory findings	42
Other amino acids	NA	When asparagine, threonine, aspartate, phenylalanine, glutamine risk of GDM is increased	43
Tryptophan metabolite, serotonin	NA	serotonin	44,45
**Fatty acids**			
Long chain polyunsaturated fatty acids and essential fatty acids	NA	Ż placental uptake	46
FFA	NA	FFA	47
**Inflammatory biomarkers**			
C-reactive protein (CRP) or highly sensitive C-reactive protein (hs-CRP)	NA	CRP, hs-CRP; not specific to pregnancy	2,5,7
	**Pregnancy period**	**Values in GDM pregnancy**	**References**
Tumour necrosis factor alpha	NA	Contradictory findings	53,57,58
Interleukin-6	NA	Contradictory findings	53,57,58
**Whole blood parameters** (not specific for pregnancy)			
Blood cell count (erythrocytes, leukocytes, neutrophils, lymphocytes and monocytes)	NA	blood cell count	12,59
MCV and MCH	NA	Ż MCV and MCH	59
Ratio of blood cells (*e.g.,* NLR or MLR)	NA	NLR, MLR	11,60-62
Platelet count	NA	platelet count	59
MPV	NA	Ż MPV	59
Ratio of pro-inflammatory T-helpers (Th1 and Th17) and anti-inflammatory cells (T regulatory cells, Treg, and Th2)	NA	ratio Th1 and Th17 to Treg and Th2	63
CD3+, CD4+, CD8+	NA	CD3+, CD4+, CD8+	65,66,67
**Novel GDM biomarkers**			
BNP	NA	Ż BNP	5
Afamin	First trimester	afamin	68
FGF21	First trimester	FGF21	69
ANGPTL8	Early pregnancy	ANGPTL8	70
Placental lactogen	NA	placental lactogen	71
Galanin	Second trimester	galanin	72
VAP1	Second trimester	VAP1	73
FABP4	Second trimester	FABP4	74
Fetuin-A or AHSG	In the first and second trimester	fetuin-A	78-82
pGCD59	Prior week 20	pGCD59	95
Extracellular vesicles	Between weeks 11 and 14	Characteristic content, further research necessary	75,77
Maternal microbiota	NA	Contradictory findings, better used as monitoring tool	85-92
PIGF	Early pregnancy	PIGF	96,97
GDM - gestational diabetes mellitus. NA - not applicable. OGTT - oral glucose tolerance test. SHBG - sex hormone-binding protein. FFA - free fatty acids. CRP - C-reactive protein. hs-CRP - high sensitive C-reactive protein. MCV - mean corpuscular volume. MCH -mean corpuscular haemoglobin. NLR - neutrophil to lymphocyte ratio. MLR - monocyte to lymphocyte ratio. MPV - mean platelet volume. Th - inflammatory T-helpers. CD - cluster of differentiation. BNP - brain natriuretic peptide. FGF21 - fibroblast growth factor 21. ANGPTL8 - angiopoietin-like protein 8. VAP1 - vascular adhesion protein 1. FABP4 - fatty acid-binding protein 4. AHSG - α2-Heremans-Schmid glycoprotein. pGCD59 - plasma glycated CD59. PIGF - placenta growth factor.			

Afamin is a vitamin E-binding protein found in human plasma, which plays a role in the antiapoptotic cellular processes associated with oxidative stress. Afamin concentrations are associated with the presence of insulin resistance among patients with polycystic ovary syndrome and it is determined by ELISA method. Koninger *et al.* in their study, which included 59 women with GDM and 51 healthy pregnant women, showed that women with GDM requiring insulin showed higher afamin serum concentrations than diet-treated patients in first trimester (*68*).

Fibroblast growth factor 21 (FGF21) is a metabolic hormone produced by the adipose tissue, liver, skeletal muscle and pancreas, and it is reported that its serum concentrations are higher under conditions of insulin resistance and obesity. FGF21 is measured using ELISA. Zibar *et al.* found that in nine healthy people, the postprandial insulin concentration correlated with basal FGF2, and Bonakdaran *et al.* found no difference in insulin resistance between healthy pregnant women and pregnant women with GDM, but women with GDM showed higher concentrations of FGF21 in first trimester (*32*, *69*). Their study included 30 pregnant women with GDM and 60 healthy ones, so we believe that more pregnant women are needed to examine true value of FGF21 as a biomarker of GDM.

Angiopoietin-like protein 8 is involved in lipid and glucose homeostasis, and its concentrations were found to be higher under conditions of GDM than normal pregnancy. Huang *et al.* showed in their study on 474 pregnant women that its determination by ELISA method in early pregnancy could be important as an early biomarker of GDM (*70*).

Placental lactogen is a peptide hormone secreted by endocrine cells, it plays a role in the regulation of insulin secretion and can be determined by ELISA methods. Recently published review shows that increased concentrations of placental lactogen are found in GDM, and that placental lactogen is also one of the biomarkers with potential applicability in predicting GDM (*71*).

Galanin is a neuropeptide that reduces insulin resistance and improves glucose uptake. In a study by Dincgez Cakmak *et al.*, values of galanin in second trimester were higher in 30 blood samples from GDM pregnant women when compared to 30 healthy pregnant women (*72*). Vascular adhesion protein 1 (VAP-1) is a glycoprotein that plays a role in inflammation and oxidative stress, whose blood values, according to Dincgez Camak *et al.’s* clinical study, are elevated in GDM compared to healthy pregnancy in second trimester (*73*). Fatty acid-binding protein 4 (FABP-4) is a protein highly expressed in adipocytes, and its concentrations in plasma are significantly increased in obese subjects and those with GDM compared to healthy pregnant women in second trimester according to one small clinical study (*74*).

Extracellular vesicles (EVs), namely exosomes sized 50-100 nm in diameter, are small vesicles released into the extracellular space with the ability to reach the blood circulation. EVs can be isolated from various biological fluids (plasma, urine, and saliva) using chromatography or ultracentrifugation-free method, as performed by Arias *et al*. in the presented clinical study and are very stable and able to protect their biological contents from degradation (*75*). They are involved in interorgan communication, and mediate the development and advancement of many disease states (*76*). It has been established that during physiological pregnancy, EV concentrations are increased (especially exosomes of placental origin, found in maternal plasma). In women with GDM, EVs carry specific cargo. The characterization of the composition of EVs in GDM between gestational weeks 11 and 14 could be used for the identification of asymptomatic women who will develop GDM, and their early treatment (*77*). The characterization can be performed using flow cytometry, which is increasingly represented in routine clinical laboratories.

Fetuin-A or α2-Heremans-Schmid glycoprotein (AHSG) is a 64 kDa glycoprotein that is produced by the liver and adipose tissue, and can be secreted by the placenta during pregnancy (*78*, *79*). It can inhibit insulin signal transduction by disrupting insulin receptor formation, which results in insulin resistance and the development of T2DM. Indeed, clinical studies have shown that increased plasma concentrations of fetuin-A and human obesity are strongly correlated with the onset of T2DM (*80*, *81*). Although several studies have yielded contradictory findings concerning fetuin-A concentrations in GDM women, in other, fetuin-A was actually significantly increased during the first and second trimesters, and was associated with an increased risk of GDM development (*80*, *82*-*84*). Therefore, fetuin-A is a good candidate biomarker for the risk of GDM. It is measured using ELISA method.

Recent studies, such as clinical study by Xu *et al.,* have determined the connection between maternal microbiota and pregnancy complications (*85*). Since the microbiome may also participate in the pathogenesis of several metabolic disorders, such as obesity, T2DM and GDM, the composition of the maternal oral or gut microbiota could be a predictive biomarker for GDM, namely, for the abundance of proinflammatory taxa (*86*, *87*). Characterization of microbiota is being performed at the RNA level (by amplification and sequencing of the bacterial 16S rRNA gene) or DNA level (by whole genome sequencing). There are a number of obstacles to introducing both methods into clinical setting, such as cost of the equipment and training of clinicians to interpret the results (*88*). Additionally, since the composition of the microbiome is determined by genotype and phenotype, and especially by diet, and since studies used different inclusion criteria (*e.g.*, Crussell *et al.* selected participants that were overweight and those at high risk for GDM), the conclusions from studies on maternal microbiota are contradictory as regards the utilization of the microbiome as a predictive GDM marker (*89*-*91*). Instead, microbiome composition may serve as a tool for monitoring maternal health, and for developing methods for the modulation of the gut microbiota in the name of maternal metabolic health (*92*).

In diabetes, the membrane glycoprotein CD59, which serves as a protective element against complement-mediated lysis, is inactivated by non-enzymatic glycation and shed from the cell membranes into the plasma. This plasma-glycated CD59 (pGCD59), measurable by flow cytometry, is used as a diabetes biomarker, and has recently been shown to be specific for GDM determination, even before the 20^th^ week of gestation (*93*-*95*). Therefore, pGCD59 could become routine test for GDM and future studies will assess the exact point at which it should be measurable in plasma.

Previous review by Huhn *et al*. highlights placenta growth factor (PIGF) as a potential early marker of GDM alone and in combination with plasma protein-A (PAPP-A), but it also showed contradictory results (*96*). A more recent study involving PIGF is by Gorkem *et al.* and, although it included 158 pregnant women and showed elevated serum PIGF values in pregnant women with GDM, study was done between 24 and 28 week of gestation and does not provide information whether PIGF is reliable early marker (*97*). Recently published small clinical study by Nuzzo *et al.* showed no difference between PIGF, soluble fms-like tyrosine kinase 1 (sFlt1) and sFlt1/PIGF ratio between GDM pregnant women and healthy ones (*98*).

Most often, these new biomarkers have been identified in single study, so it is difficult to comment on whether they are indeed a good early GDM biomarker. Additional research is needed for each individual biomarker and the determination of exact cut-off value that would separate healthy pregnant women and women with GDM, at the earliest possible stage of pregnancy. Positive side is that majority of new biomarkers are determined by immunochemical ELISA method and are suitable for routine measurement in clinical laboratories although there is no reference method or sample for any of them, and the immunochemical methods are not comparable. Therefore, special importance should be given to the interpretation of the obtained values, and this is an aggravating circumstance when determining cut-off values or reference intervals.

## Conclusion

Gestational diabetes mellitus is an increasingly common global health problem, and its early diagnosis remains a challenge since none of the existing biomarkers show high specificity for GDM and therefore, the diagnosis is made in the second trimester. Early diagnosis and treatment are crucial because inadequate intrauterine conditions for growth and development in the critical period of foetal life have an impact on foetal programing, and can induce disease in the early postnatal period as well as chronic disease later in life.

Our literature search for the new early biomarker of GDM resulted with few possible candidates: afamin, angiopoietin-like protein 8, characterization of the composition of EVs, fetuin-A, FGF21, pGCD59. Each of them has increased or altered concentrations in early stages (mainly first trimester) of GDM pregnancies compared to healthy pregnancies and they are measurable in plasma or serum using laboratory techniques, which could be at some point automated. Future, more extensive, studies are needed to measure and calculate their decision threshold and to evaluate their clinical usefulness, availability of a referent method and referent material as well as analytical sensitivity and specificity of the methods used.
